# Pelvic floor muscle training as an adjunct to prolapse surgery: a randomised feasibility study

**DOI:** 10.1007/s00192-013-2301-x

**Published:** 2014-02-06

**Authors:** Doreen McClurg, Paul Hilton, Lucia Dolan, Ash Monga, Suzanne Hagen, Helena Frawley, Lucy Dickinson

**Affiliations:** 1 Nursing, Midwifery and Allied Health Professions Research Unit, Glasgow Caledonian University, Glasgow, G40BA UK; 2 Directorate of Women’s Services, Royal Victoria Infirmary, Newcastle upon Tyne, UK; 3 Belfast Health and Social Care Trust, Belfast, UK; 4 Southampton University Hospitals, Southampton, UK; 5 University of Melbourne, Melbourne, Australia

**Keywords:** Prolapses, Pelvic floor muscle training, Recurrence, Symptoms, Trial

## Abstract

**Introduction and hypothesis:**

There is evidence that in nonsurgical populations, pelvic floor muscle training (PFMT) and lifestyle advice improves symptoms and stage of pelvic organ prolapse (POP). Some women, however, require surgery, after which de novo symptoms can develop or additional surgery is required due to recurrence. Robust evidence is required as to the benefit of perioperative PFMT in the postsurgery reduction of symptoms and POP recurrence. The aim of this study was to assess the feasibility of and collect pilot data to inform sample size (SS) calculation for a multicentre randomised controlled trial (RCT) of perioperative PFMT following surgical intervention for POP.

**Methods:**

Fifty-seven participants were recruited and randomised to a treatment group (one pre and six postoperative PFMT sessions) or a control group (usual care). The primary outcome measure was the Pelvic Organ Prolapse Symptom Score (POP-SS) at 12 months; secondary outcome measures included measurement of prolapse, the pelvic floor and questionnaires relating to urinary and bowel incontinence. All outcomes were measured at 0, 6 and 12 months.

**Results:**

Information on recruitment, retention and appropriateness of outcome measures for a definitive trial was gathered, and data enabled us to undertake an SS calculation. When compared with the control group (*n* = 29), benefits to the intervention group (*n* = 28) were observed in terms of fewer prolapse symptoms at 12 months [mean difference 3.94; 95 % confidence interval (CI) 1.35–6.75; *t* = 3.24, *p* = 0.006]; however, these results must be viewed with caution due to possible selection bias.

**Conclusion:**

With modifications to design identified in this pilot study, a multicentre RCT is feasible.

**Electronic supplementary material:**

The online version of this article (doi:10.1007/s00192-013-2301-x) contains supplementary material, which is available to authorized users.

## Introduction

Pelvic organ prolapse (POP) is a common condition characterised by symptomatic descent of the vaginal walls and/or uterus or vaginal vault from their normal anatomical position [1]. Estimates of prevalence of POP in the general population are variable; however, in a sample of 2,979 women between 45 and 86 years of age, reported in 2010, 21 % were found to be symptomatic [2]. Women with prolapse may present with vaginal, bladder, bowel, musculoskeletal and sexual symptoms. Those women with symptomatic POP who fail or decline conservative management are candidates for surgery, with 80–90 % being undertaken by the vaginal route [3]. Surgery will attempt to restore normal vaginal compartments and re-establish or maintain normal urinary, rectal and sexual functions whilst attempting to minimise adverse effects [4]. Women have an 11 % risk of undergoing at least one surgical intervention for POP or incontinence by the age of 79 years, and due to our increasing ageing population, it has been suggested that this risk might double in the next 30 years [5, 6]. The long-term outcome following surgical correction of POP is poor, and in a prospective study, 41 % of women had recurrence of POP at 5 years, and 10 % of women had undergone a repeat POP operation within 5 years of their index operation [7].

The pelvic floor muscles (PFMs) are an integral component of the support mechanism of the pelvic organs. Bø reviewed basic research and case–control studies and put forward two hypotheses [8]:

1. Women can build up muscle tone and structural support of the PFMs through regular strength training over time.
2. Women can learn to contract their PFMs consciously before and during an increase in intra-abdominal pressure and will continue to make such contractions as a behavioural modification in order to prevent descent of the pelvic contents.

In addition, a study by Braekken et al. [9] demonstrated elevation of the pelvic organs and reduction in the levator hiatal area after PFMT and assumed that PFMT can be used to prevent POP. We therefore hypothesised that women who have been taught and encouraged to perform PFM exercises, will have established a coordinated muscular support mechanism to withstand increases in intra-abdominal pressure and for that reason they will experience fewer POP symptoms after surgery and have a reduced rate of POP recurrence in the long term.

In 2008, a UK survey of members of the Association of Chartered Physiotherapists in Women’s Health (ACPWH) was undertaken [10]. It was evident that there is wide variation in practice amongst physiotherapists in terms of the intervention they provide for women undergoing prolapse surgery. Respondents reported that there was a lack of evidence for their intervention and many felt dissatisfied with this situation. International surveys of physiotherapy practice following surgery for POP have reported similar findings [10–13].

Some studies in nonsurgical populations support PFMT as a treatment in its own right in women with POP [9, 14]. However, a review of the literature relating to perioperative physiotherapy in benign gynaecological conditions only identified two small randomised controlled trials (RCT) [15, 16] on the subject, neither of which reported on POP symptoms. Jarvis et al. reported on a two-group RCT (*n* = 30 per group) with results suggesting a significant benefit in women who received treatment; however, conclusions were limited due to the short length of follow-up and a lack of prolapse-specific outcome measures [15]. Frawley et al. reported a two-group RCT (*n* = 25 per group) that showed no significant differences between groups [16]. The paper presented here reports on a study, including a pilot trial, to evaluate the feasibility of a multicentre RCT to assess the effectiveness of perioperative PFMT and lifestyle advice for women who are undergoing surgery for POP and is the first to use a POP-specific questionnaire as an outcome measure; this was designated as the SUrgery and Physiotherapy for prolapsE Research feasibility study (SUPER) study, the aims of which were:

1. To develop the methods and assess the feasibility of a multicentre RCT of perioperative PFMT and lifestyle advice for women undergoing surgical intervention for POP. Information on recruitment, retention and suitability of outcome measures specific to POP and associated symptoms were sought.
2. To collect pilot trial data to inform sample-size calculations and optimal health economic methods in preparation for undertaking a multicentre pragmatic RCT.

## Methods

Ethical approval was granted at the three centres (West of Scotland Rec 5, 15 September 2010, 101001/45; Newcastle Ref 5494, 23 November 2010; Southampton RHM O & G0177, 19 January 2011; Belfast Ref 10192DMcC-SC, 10 June 2011). Our target sample size was 30 per group. This number was based on (1) the prediction (based on patient numbers) that each of the three centres could recruit 20 participants (ten in each group) in the time provided; (2) our experience of recruitment in similar pilot studies; and (3) that this number would be sufficient to test out recruitment, randomisation, intervention, follow-up processes and provide data for a sample size calculation [17]. Women who attended the gynaecological clinic and for whom primary surgery was recommended due to their POP symptoms were asked to participate in the trial. If they were interested and completed the consent form, the patient was randomised using a remote computer programme that then sent an email with the group allocation of the participant to the researcher, using the identification code initially assigned to the participant. Minimisation variable was included for each centre. Randomisation was to the treatment (TG) or the control (CG) group. TG patients (*n* = 28), received perioperative PFMT by a women’s health physiotherapist [treatment physiotherapist (TP)]. This standardised intervention included:

- A preoperative appointment
  A standardised history was taken. Anatomy and function of PFMs was discussed and types of prolapse described using diagrams and a model of the pelvis. The proposed surgical procedure was discussed and information provided about recovery and return to normal activities of daily living (ADL). Women were taught (by digital palpation) how to correctly contract their PFMs and how to precontract against increases in intra-abdominal pressure (the knack) [18]. At this visit, women were advised to do three sets of ten maximum contractions (up to 10-s hold) per day with 4-s rest between sets, following by a 1-min rest followed by ten fast contractions.
- Postdischarge
  During the postdischarge week, the TG were mailed a lifestyle advice leaflet specifically devised for use in the SUPER study. This contained information on types of prolapse; advice on recovery during the early postoperative phase; and advice on avoidance of constipation, heavy lifting, high-impact exercise and correct technique of defaecation and bracing before coughing and lifting. The women in this group were also telephoned once during this week by a member of the research team to answer questions relating to their recovery.
- Outpatient appointments
  During the first outpatient session (at week 6), a brief history of postoperative recovery was recorded, and a repeat vaginal examination assessed the participant’s ability to contract their PFMs. Five further weekly physiotherapy outpatient appointments within a period of 12 weeks were provided. An individualised home exercise programme was prescribed, with progression dictated by improvement in PFM function. To facilitate PFM contraction and encourage adherence, physiotherapists were allowed to use adjuncts such as biofeedback, electrical stimulation and exercise balls, as per their usual practice. Symptom changes, compliance with lifestyle advice and changes in PFM strength were recorded at each subsequent consultation, and the content of the home exercise programme was adjusted accordingly.

Control group (CG) patients (*n* = 29), were mailed the same lifestyle advice leaflet as those in the TG. This group did not see a research physiotherapist and had no planned contact with the hospital until the follow-up gynaecology appointments relating to their surgery.

In order to standardise the physiotherapy intervention, all study sites were visited by the study chief investigator, who gave instruction in the delivery of the lifestyle advice, content of the appointments, use of diaries and standardised leaflet and clinical documents. All study physiotherapists were experienced in the delivery of PFMT. In all other respects, study groups received postoperative advice as per local practice; e.g. a locally approved advice leaflet and/or a brief discussion with a women’s health physiotherapist or nurse.

### Outcome measurement

Both groups of women completed postal questionnaires at three times points: baseline (immediately prior to randomisation), and at 6 and 12 months after randomisation. The outcome of primary interest was prolapse symptom severity as measured by the Pelvic Organ Prolapse Symptom Score (POP-SS). This is a validated, self-completed, seven-item questionnaire scored from 0 to 28, with higher scores indicating more symptoms [19, 20]. In addition, participants completed the International Consultation on Incontinence Questionnaire Urinary Incontinence Short Form (ICIQ-UI SF) [21], the ICIQ Bowel Symptom (ICIQ-BS) [22], POP/Urinary Incontinence Sexual Questionnaire 12 (PISQ-12) [23], and the Short Form Health Questionnaire of 12 questions (SF-12) a generic measure of perceived health [23, 24]. An outcome physiotherapist was trained at each centre to assess the degree of prolapse using the POP Quantification (POP-Q) scale, and all were experienced in undertaking digital PFM evaluation using the PERFECT [P representing power (or pressure, a measure of strengthusing a manometric perineometer), E = endurance,R = repetitions, F = fast contractions, and ECT = everycontraction timed] and Modified Oxford scales. The former is a feasible and reliable assessment for physiotherapists to undertake [25], and the latter has high interexaminer and test–retest reliability [26]. The outcome therapist was blinded to group allocation, and the same centre therapist undertook all assessments at all time points (0, 6 and 12 months) with the women in the modified dorsal lithotomy position

An exit questionnaire was developed and sent to participants with the final questionnaires. This had six sections with two to four questions per section. Each question contained Yes/No and Can’t Remember boxes, with space for any additional comments. Sections included feedback on prestudy information, outcome measures and assessment burdensomeness and intervention acceptability. Results of this questionnaire will also feed into the proposed RCT.

### Data analysis

As this was a pilot study, many outcomes were related to the feasibility of undertaking the RCT; e.g. willingness of clinicians to recruit and of participants to be randomised, number of eligible patients and follow-up rates, response rates, compliance and standard deviation (SD) for our primary outcome measures (OCM) to allow sample size calculation for the main RCT. Comparison of treatment effectiveness was a secondary outcome, so statistics are largely descriptive; statistical testing was kept to a minimum. Tables showing means and SDs (and other summary statistics as appropriate) for both groups were produced. Unpaired *t* tests or nonparametric equivalents were employed to test for differences in continuous outcomes between groups, and mean differences with 95 % confidence intervals (CI) are reported where appropriate. Data were blinded before analysis on an intention-to-treat basis and stored in accordance with the UK Data Protection Act 1998.

## Results

**Fig. 1:**
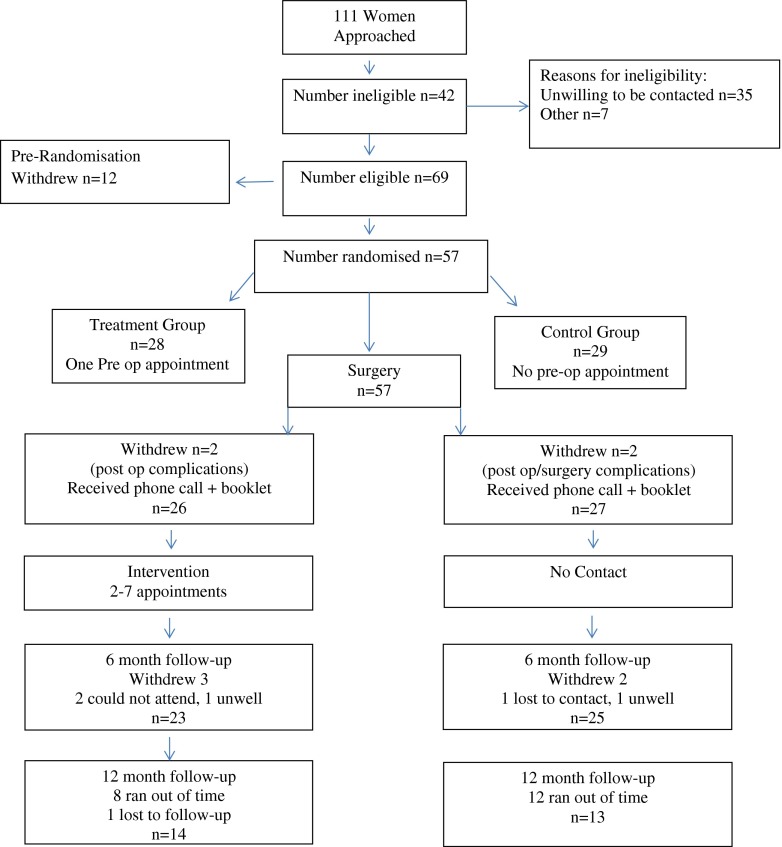
Consolidated Standards of Reporting Trials (CONSORT) flow diagram

Sampling and recruitment was undertaken as shown in the Consolidated Standards of Reporting Trials (CONSORT) flow chart (Fig. 1). Fifty-seven participants were randomised: 28 to the TG and 29 to the CG. Median age was 60 (range 35–80) years, average number of births was 2.19 (SD 0.83), mean body mass index (BMI) was 27 (SD 3.0), mean duration of prolapse symptoms was 17 months and the most common prolapse stage was II, with 50 % being anterior prolapse. Both groups demonstrated similar symptom severity at baseline (Table 1), except that the TG was more troubled by bowel dysfunction compared with the CG. When comparing POP-Q assessment at baseline, those in the TG were more likely to demonstrate posterior prolapse. Of patients who were approached and were eligible, approximately 50 % agreed to participate. No adverse events were reported during the follow-up period.

**Table 1:** Summary of prolapse-related quality of life (QoL) scores at baseline and 6 months for treatment (TG) and control (CG) groups (all centres)

Measurement	TG baseline, mean (SD)	CG baseline, mean (SD)	TG, mean (SD)	CG, mean (SD)	Between-group difference in change, (95 % CI), *t* value, *p* value
POP-SS Lower scores indicate better QoL	13.40 (6.57)	13.44 (5.69)	3.90 (4.54) *t* = 6.70 *p* = 0.00	3.72 (3.89) *t* = 7.51 *p* = 0.00	(−2.561 to 2.192) −0.156 0.876
ICIQ-UI; lower scores indicate better QoL	6.21 (4.68)	6.13 (6.62)	2.75 (3.67) *t* = 3.618 *p* = 0.01	3.32 (3.47) *t* = 2.062 *p* = 0.05	(−1.48 to 2.97) −0.050 0.96
ICIQ-BS; lower scores indicate better QoL	16.92 (6.08)	13.55 (3.07)	11.59 (3.83) *t* = 4.42 *p* = 0.00	12.42 (3.38) *t* = 1.16 *p* = 0.25	(−1.26 to 2.93) −2.64 0.11
SF-12; higher score indicates better QoL	33.57 (3.55)	35.14 (6.73)	46.43 (13.35) *t* = 4.02 *p* = 0.01	49.12 (19.12) *t* = 3/268 *p* = 0.03	(−6.97 to 12.35) 0.559 0.579

*POP-SS* Pelvic Organ Prolapse Symptom Score, *ICIQ-BS* International Consultation on Incontinence Questionnaire Bowel Symptom, *SF-12* Short Form Health Survey of 12 items, *SD* standard deviation, *CI* confidence interval

### Retention and adherence

One centre recruited and retained participants above target (target *n* = 20: *n* = 27). There were delays in recruiting to time and target at two centres, which meant that no participants at one site and only four at the other had 12-month follow-up data; thus, overall results at 12 months are weighted to one unit. The questionnaire response rates at 6 months were TG = 23/28 (82 %) and CG = 25/29 (86 %). Completion of the questionnaires at 12 months was 14/28 (50 %) in the TG and 13/29 (44 %) in the CG; due to the recruitment difficulties, 20 participants failed to reach the 12-month stage, and one was lost to follow-up. Of those in the centre that recruited to target, TG = 11/14 (92 %) and CG = 12/13 (78 %) returned the questionnaires at 12 months (Table 2). Attendance at the POP-Q and PFM review appointment was TG = 26/28 (92 %), CG 26/29 (89 %) at baseline; TG = 14/28 (50 %) and CG = 11/29 (28 %) at 6 months; and TG =  5/28 (18 %) and CG =  5/29 (17 %) at 12 months (Tables 3 and 4). Adherence of therapists to the intervention protocol appeared high (as per information accessed from assessment and treatment forms). Adherence to home exercise was assessed using a home exercise diary. Although overall the compliance of completion of this diary was poor, 8/28 (28 %) completed all diaries, and those that were completed recorded good compliance.

**Table 2:** Adherence to completion of outcome measure questionnaires

All questionnaires	Treatment group	Control group	Newcastle (*n* = 27); CG/TG
Baseline	28 (100 %)	29 (100 %)	13/13; 14/14
6 months	23 (82 %)	25 (86 %)	12/13; 12/14
12 months	14 (50 %)	13 (44 %)	12/13; 11/14

**Table 3:** Pelvic organ prolapse (POP) stage as measured using the POP Quantification (POP-Q) at baseline and 6 and 12 months

Stage of Prolapse	Baseline	Baseline	6 month	6 month	12 month	12 month
	TG (*n* = 26)	CG (*n* = 26)	TG (*n* = 14)	CG (*n* = 13)	TG (*n* = 5)	CG (*n* = 5)
Stage 0	0	0	10	11	5	4
Stage I	2 (7 %)	2 (7 %)	4	2		1
Stage II	18 (64 %)	15 (57 %)				
Stage III	5 (20 %)	9 (34 %)				

*TG* treatment group, *CG* control group

**Table 4:** Pelvic floor muscle assessment

Measure	TG baseline	CG baseline	TG 6 months	CG 6 months	TG 12 months	CG 12 months
	*n* = 25	*n* = 26	*n* = 14	*n* = 15	*n* = 5	*n* = 5
	mean (SD)	mean (SD)	mean (SD)	mean (SD)	mean (SD)	mean (SD)
Power	2.56 (0.935)	2.81 (1.021)	3.57 (0.646)	2.87 (1.060)	3.50 (0.654)	2.36 (0.809)
Endurance (s)	7.13 (3.123)	6.6 (3.279)	8.43 (2.441)	7.30 (2.720)	7.00 (2.453)	7.27 (2.240)
Repetitions	6.41 (2.501)	5.88 (3.0470)	7.93 (2.018)	6.62 (2.329)	10 (2.345)	7.91 (2.256)
Hold with cough: Yes/N	12/25 (48 %)	13/26 (50 %)	13/14 (92 %)	10/15 (66 %)	5/5 (100 %)	3/5 (60 %)

*TG* treatment group, *CG* control group, *SD* standard deviation

### Symptoms

The primary symptom outcome measure was the POP-SS completed by women in their questionnaires. Both groups reported similar improvement from baseline to 6 months. Similar improvements were also reported in the ICIQ-UI, ICIQ-BS and SF-12 from baseline to 6 months (Table 1). There was a significant difference in POP-SS and SF-12 scores between groups at 12 months [mean difference (MD) 3.94545; 95 % CI 1.358–6.750; *t* = 3.248, *p* = 0.006); MD −6.88333; 95 % CI −11.344 to −2.431, *t* = −3.218, *p* = 0.004] (Table 5)

**Table 5:** Summary of questionnaires at baseline and 6 and 12 months (Newcastle only)

Measurement	Treatment group baseline	Control group baseline	Treatment group	Control group	Treatment group	Control group	Between-group difference:
			6 months	6 months	12 months	12 months	0–12 months
	mean/SD	mean/SD	mean/SD	mean/SD	mean/SD	mean/SD	(95% CI), *t*, *p* value
POP-SS	13.38 (5.76)	14.69 (5.40)	3.27 (4.50)	5.41 (5.96)	2.45 (2.42)	6.40 (3.40)	(1.35 to 6.75) *t* = 3.24 *p* = 0.06
ICIQ-UI	6.30 (7.69)	6.30 (4.47)	3.54 (4.69)	3.84 (3.69)	1.30 (1.60)	3.23 (3.60)	(−0.33 to 4.18) *t* = 1.75 *p* = 0.92
ICIQ-BS	14.53 (5.36)	13.92 (3.64)	12.90 (5.02)	12.85 (3.95)	11.40 (4.52)	11.38 (3.01)	(−4.22 to −3.00) *t* =−0.348 *p* = 0.731
SF-12	35.20 (0.92)	34.83 (6.01)	42.82 (3.68)	40.20 (5.20)	42.58 (3.60)	35.70 (6.29)	(−11.34 to −2.43) *t* =−3.218 *p* = 0.04
PISQ	42.69 (23.90)	38.53 (19.91)	36.23 (26.56)	36.84 (22.69)	31.07 (23.19)	29.38 (17.76)	(−13.65 to 21.96) *t* = 0.481 *p* = 0.635

*POP-SS* Pelvic Organ Prolapse Symptom Score, *ICIQ-BS* International Consultation on Incontinence Questionnaire Bowel Symptom, *SF-12* Short Form Health Survey of 12 items, *PISQ* POP/Urinary Incontinence Sexual Questionnaire, *SD* standard deviation, *CI* confidence interval

.

### Outcomes recorded by the outcome physiotherapist

#### POP-Q (Table 3)

Preoperatively, 51 women were assessed, 33/51 (65 %) with stage II and 14/51 (27 %) with stage III POP. At 6 months postoperatively, the numbers attending for assessment were TG =14/28(50 %) and CG = 11/29(28 %); at 12 months, 5/28 (18 %) and 5/29 (17 %) attended. No woman in either group demonstrated prolapse greater than stage 1 at either 6 or 12 months.

#### Pelvic floor muscle assessment (Table 4)

Participants in the TG recorded a mean improvement in PFM strength of 1.0 in the Oxford classification, whereas those in the CG recorded an improvement of 0.06. Holding time and number of repetitions also increased more in the TG than the CG from baseline to 6 months. At 6 months, 13/14 (92 %) of the TG could contract and hold the contraction whilst coughing, compared with 10/15 (66 %) in the CG (Table 4).

### Exit questionnaire

The exit questionnaire was completed and returned by 22 participants (11 from each group). Those in the TG reported that they found being in the study was useful, agreed that the number of visits was about right, and would take part again, with eight reporting continuation of their PFM exercises. Of those in the CG, five expressed disappointment about being in the CG; 7/11 had sought additional information from web sites/books or local Pilates-type classes led by a eoman’s health physiotherapist, and 8/11 said they were dissatisfied with the information on postoperative recovery/PFMT they received, i.e. usual care.

### Feedback from outcome (OP) and treatment (TP) physiotherapists

TPs reported that most participants were very pleased with the additional support and felt it helped with their recovery. Keeping track of the surgery dates was difficult at two centres, as the physiotherapists did not have direct access to the surgery lists.

### Sample size calculation

Study results provided us with the information to undertake a sample size calculation for a future pragmatic multicentre RCT of effectiveness using the women’s symptoms, as measured by the POP-SS as primary outcome. The minimally important clinical difference (MICD) in the POP-SS is 1.5 [20]. Thus, we would want a future trial to have sufficient statistical power to detect a difference between groups of 1.5 in the POP-SS, as women have indicated that this equates to a meaningful change in symptoms. Using the SD of the POP-SS at 12 months from our pilot trial (SD = 4) as an estimate of likely variability in the outcome measure, this would suggest a sample size of 150 per group to achieve 90 % power at the 5 % level of significance. Allowing for 30 % attrition, this would bring the sample size to 214 per group. This calculation, however, is primarily based on evidence from the one centre that had data at 12 months (as explained earlier, the other two centres did not recruit in time to allow completion of the 12-month data), so this calculation may overestimate participation. However, as discussed later, many lessons have been learned from undertaking this pilot study that would enhance recruitment; but based on our findings, we estimate we would need 20 sites recruiting 20 patients each over a period of 2 years.

## Discussion

To our knowledge, this is the first pilot RCT comparing adjunctive perioperative PFMT and lifestyle advice to standard care following surgery for POP, using prolapse symptoms as the primary outcome measure. We achieved our objective of demonstrating that such a clinical trial was feasible and believe the recruitment of our proposed number of centres with adequate TP and OP staff is possible: e.g. in a trial of outpatient PFMT as treatment for POP, we recruited 23 centres in the UK at which we had two physiotherapists involved; many of these centres also undertake gynaecological surgery [25]. The main difficulty with this study was slow recruitment at two centres, and most 12-month data came from one centre. This centre, in which processes were robust and staff were established, recruited to time and target. However, at one other centre, the principal investigator (PI) changed National Health System (NHS) location, which delayed commencement for a considerable time; at the other centre, the TP was on long-term sick leave with no replacement. Whereas similar events may occur in the RCT, we learned from this experiences and would more quickly withdraw centres who were not recruiting to target and have additional sites prepared to participate. The study setup time and recruitment period would also need to be extended, which would give some flexibility for research approvals and training to be completed. In addition, at each centre, we would have a dedicated research nurse/allied health profession who would approach potential participants previously identified by screening patient notes. This would reduce the burden on local PIs during busy clinics. Approximately 50 % of eligible patients were willing to be randomised between treatment and control arms, and those in the TG attended 80 % of treatment sessions and appeared adherent to the prescribed exercises and advice. Overall, completion of outcome measures at 6 months was 84 %. Completion at 12 months was 85 % of the centre that recruited to time. This level of completion of postal questionnaires is similar to other studies we have completed; however, we will put in place strategies to ensure we maintain contact with participants in the long term; e.g. we have experience in keeping in contact with patients long term by using such details as a relatives’ or friends’ contact (supplied with permission). Attendance at follow-up appointments for PFM assessment and POP-Q were disappointing and not equivalent to some other studies; e.g. the Pelvic Organ Prolapse Physiotherapy (POPPY) study had a 77 % (365/477) attendance for the 6-month review [14], and the Pelvic Floor Muscle Training to Prevent Pelvic Organ Prolapse in Women (PREVPROL) study 70 % at 1 year (280/400) (personal communication). In the definitive trial, we intend to apply for funding to cover travel and parking expenses of participants attending for follow-up assessments and be more proactive in organising appointments at a more suitable time for woman who may have returned to work, with the possible use of incentives on completion. Long-term follow-up would be imperative in the proposed RCT, and we would apply for funding to allow us to access NHS data on these patients for at least 10 years, as preventing POP recurrence necessitating further NHS consultation and/or surgery is a primary aim. In addition, we could obtain consent to send a questionnaire to women at 5 and 10 years to capture health, lifestyle, and activity.

Our QoL and symptom questionnaires appear to be sensitive within this population and were not overburdensome to participants. There is the option of using perineometry or transperineal ultrasound (US) rather than or as well as digital vaginal assessment of PFMs. The PERFECT and Modified Oxford scale we used is widely taught in the UK and has been shown to be reliable, as well as providing an opportunity to correct technique [26]. The Peritron perineometer is a simple and reliable objective measure of PFM strength via vaginal squeezing pressure measurement [27], although Ferreira et al. recommended that for research purposes, the same examiner assess and reassess patients for both the Peritron and the Oxford grading scale [28]. Transperineal US demonstrates reliability and may also provide evidence of effect [29]. Monitoring adherence to PFM exercise is important both in the short- and long term. Home exercise diaries are notorious for lack of completion and/or lack of fidelity. We are reviewing the use of diaries within both research and clinical practice and looking at electronic means of recording exercise adherence with the potential to identify the optimal method for future trials.

To undertake such a study would be relatively expensive in terms of NHS excess treatment costs. To cover the additional visits, it would cost approximately £280 per patient in the treatment arm. Research support costs for outcome measures such as pelvic floor assessment and POP-Q would also be required. It is therefore important that within the main trial an economic evaluation is undertaken to establish whether this intervention is likely to be cost effective.

It would appear that patients in the treatment group reported fewer symptoms at 12 months than those in the control group (Table 5). However, we suggest that this finding be treated with caution, as it is based primarily on responses from one site. We recognise that opinions vary as to validity of reporting statistical analysis in an external pilot study; however, we wanted to look at the size difference between groups to see whether data would give us at least an indication of whether the intervention is potentially effective. It may be a real effect observed at one good site, which would not be replicated more generally if there was a wider rollout and therefore does not preclude the need for a larger pragmatic trial to establish effectiveness. Moreover, in order to establish benefit, a 5- to 10-year follow-up would be required, as second repair procedures occur principally within this time frame [30].

This pilot study and the literature also raise several questions. For example, how much benefit from, and to what extent do patients adhere to, lifestyle advice, and what advice should patients receive following POP surgery? Our study supports findings from clinician surveys that unclear and conflicting advice about the timing of return to activities of daily living and sport is often provided to patients [9–13]. In addition a review by Murphy et al. [31] reported limited data to guide many other aspects of postoperative care. A literature review undertaken in 2013 reported that no randomised or prospective cohort studies that reported the association between postoperative activity and surgical success after pelvic floor repair could be identified [32], although Barber et al. published a protocol on two types of vaginal-vault surgery and PFMT [33]. Before commencing a definitive trial, efforts should be made to define the most appropriate advice for women following POP surgery. Consensus methods, such as the Delphi survey technique, could be used to transform opinion into group consensus [34].

## Conclusion

The aim of this study was to determine the feasibility of a future definitive trial; hence, we should not draw any definite conclusions about treatment effect. We believe that with modifications to design, such an RCT is practical and would provide evidence from which clinical guidance could be developed regarding the place of perioperative PFMT to limit POP recurrence.

## Electronic supplementary material

Below is the link to the electronic supplementary material.

ESM 1 (DOCX 18.2 kb)
