# Supplementary material for: Pelvic floor muscle training as an adjunct to prolapse surgery: a randomised feasibility study
Source: Int Urogynecol J. 2014 Feb 6;25(7):883–91. doi: 10.1007/s00192-013-2301-x (PMC4057627; doi:10.1007/s00192-013-2301-x)
Supplement: Supplementary file 1 — (DOCX 18.2 kb) [file 192_2013_2301_MOESM1_ESM.docx]

**Table 1 Summary of Questionnaires at baseline and 6 months (All centres)**

| **Measurement** | **Treatment group baseline**  **Mean/SD** | **Control group baseline**  **Mean/SD** | **Treatment group**  **6 months**  **Mean/SD** | **Control**  **group**  **6 months**  **Mean/SD** | **Between group difference**  **95% CI, t, p value** |
| --- | --- | --- | --- | --- | --- |
| **POPSS**  **Lower scores indicate better QoL** | 13.40  (6.57) | 13.44  (5.69) | 3.90 (4.54)  t=6.705  p=0.000 | 3.72 (3.89)  t=7.514  p=0.000 | (-2.561,2.192)  t=-.156  p=.876 |
| **ICIQ-UI**  **Lower scores indicate better QoL** | 6.21  (4.68) | 6.13  (6.62) | 2.75 (3.67)  t=3.618  p=0.001 | 3.32 (3.47)  t=2.062  p=0.050 | (-1.483,2.978)  t=-.050  p=0.960 |
| **ICIQ-BS**  **Lower scores indicate better QoL** | 16.92  (6.08) | 13.55  (3.07) | 11.59 (3.83)  t=4.427  p=0.000 | 12.423 (3,38)  t=1.160  p=0.257 | (-1.266,2.930)  t=-2.645  p=0.11 |
| **SF12**  **Higher score indicates better QoL** | 33.57  (3.55) | 35.14  (6.73) | 46.43 (13.35)  t=4.02  p=0.001 | 49.12 (19.12)  t=3/268  p=0.003 | (-6.979,12.350)  t=.559  p=.579 |
